# Asymmetry in college basketball players: change of direction performance in shuffle movement and 505 test

**DOI:** 10.3389/fphys.2025.1587719

**Published:** 2025-04-30

**Authors:** Peng Wang, Mengde Lyu, Na Geng, Zirun Wu, Xinran Ren, Žiga Kozinc, Rafael Kons, Nejc Šarabon, Xiangjun Miao

**Affiliations:** ^1^ Chinese Basketball College, Beijing Sport University, Beijing, China; ^2^ School of Athletic Performance, Shanghai University of Sport, Shanghai, China; ^3^ School of Physical Education, Anhui Normal University, Wuhu, China; ^4^ Faculty of Health Sciences, University of Primorska, Koper, Slovenia; ^5^ Department of Physical Education, Faculty of Education, Federal University of Bahia, Salvador, Brazil; ^6^ Andrej Marušič Institute, University of Primorska, Koper, Slovenia

**Keywords:** interlimb, physical tests, vertical jump, movement performance, basketball player

## Abstract

**Introduction:**

This study aims to 1) examine the difference and asymmetry in change of direction in shuffle movement (CoDS) performance among basketball players; 2) assess the relationship and directional agreement between asymmetry scores of CoDS, 505, and four single leg jump tests.

**Methods:**

Forty-two college basketball players performed three trials of the CoDS and 505 tests for each leg, with the fastest performance used for the final analysis. Single leg countermovement jumps, lateral jumps, broad jumps, and drop jumps were also performed for both legs. The dominant (D) limb was defined as the side that performs better, while the non-dominant (ND) limb was the opposite. Paired-sample t-tests were conducted to compare differences in D and ND across all performances. The Kappa coefficient was used to assess consistency in asymmetry direction. Spearman’s ρ correlations were applied to examine relationships between normally and non-normally distributed performance and inter-limb asymmetry data.

**Results:**

Significant differences were found in CoDS (Asymmetry Score = 3.66 ± 2.74, ES = 0.49, *p* < 0.01) and 505 (Asymmetry Score = 2.54 ± 2.28, ES = 0.34, *p* < 0.01) performance between D and ND limbs. However, there was no significant correlation between the asymmetry scores in CoDS and 505 (*p* > 0.05). The Kappa coefficients between CoDS, 505 and single leg jump tests showed poor to slight agreement (Kappa range from −0.07–0.22) regarding asymmetry direction.

**Conclusion:**

These results suggest that practitioners should specialize in assessing CoD asymmetry in both forward-backward and lateral movement performance through 505 and and CODS test.

## 1 Introduction

Basketball is a sport that highly relies on multidirectional movement capabilities (Stojanović et al., 2018). During a game, athletes frequently perform rapid changes of direction (CoD), lateral movements, jumps, accelerations, and decelerations (Taylor et al., 2017), placing high demands on the balanced development and coordination of both limbs (Ding et al., 2024). For example, players must respond quickly to offensive moves by shuffling laterally (Lyu et al., 2025b), allowing them to defend effectively and gain a subsequent advantage (Leidersdorf et al., 2022). Inter-limb asymmetry refers to the differences between an individual’s left and right limbs in terms of strength, flexibility, range of motion, coordination, or other motor abilities (Bishop et al., 2023). This asymmetry often manifests as superior function or performance in one limb over the other, potentially resulting in imbalanced movement patterns during athletic activities (Maloney, 2019). For instance, some athletes may favor one limb when jumping, changing direction, or running, which could impact overall performance (Bishop et al., 2018a). Therefore, assessing inter-limb asymmetry in various performance metrics is crucial to ensure that athletes maintain optimal performance on the court (Bishop et al., 2018b; Loturco et al., 2019).

CoD performance is crucial for basketball players, as it directly impacts their agility, reaction speed, and overall performance on the court (Klusemann et al., 2013). Previous studies have examined 505 CoD performance in athletes and analyzed inter-limb asymmetry in 505 performance, which indicated that the 505 CoD asymmetry score ranged 2.60% – 3.27% in different sessions, which indicates the presence of interlimb asymmetry in athletes’ CoD ability in the forward and backward directions (11). However, the full distance and movement pattern of the 505 test may not entirely align with the actual demands of basketball sport, where players frequently engage in extensive lateral movement. As noted by Mohr and Federolf, athletes performing lateral movement execute actions such as shuffling, 180° CoD, and side-cutting maneuvers (Mohr and Federolf, 2022), with basketball players often completing lateral movements using a shuffling technique (Leidersdorf et al., 2022). Additionally, a systematic review by Taylor et al. on movement patterns in team sports found that basketball players perform up to 450 lateral movements per game (Taylor et al., 2017). Consequently, assessing CoD ability and asymmetry in lateral movements among basketball players is essential for comprehensively understanding their specific skills, as CoD ability may underpin movement proficiency and defensive ability. Previous studies have used various lateral shuffle tests, such as the submaximal lateral shuffle test and lateral side-step tests (McCormick et al., 2016; Maurus et al., 2019), but these tests are primarily aimed at measuring the maximum speed of lateral shuffle movements and do not reflect CoD ability during lateral movements. Given the widespread use of the 505 test in CoD assessments (Nimphius et al., 2016; Balsalobre-Fernández et al., 2019; Bishop et al., 2022b), it may be worth exploring whether the principles of the 505 test could be applied to measure CoD performance in lateral movements.

Single leg jump ability is equally important for basketball players, with single leg jump tests widely used to detect inter-limb asymmetry due to their multi-directional applicability and ease of execution (Bishop et al., 2023). Common tests include the single leg countermovement jump (SLCMJ), single leg lateral jump (SLLJ), single leg broad jump (SLBJ), and single leg drop jump (SLDJ) (Madruga-Parera et al., 2021; Bishop et al., 2022b). Prior studies have shown a relationship between single leg jump asymmetry and CoD ability. For example, Bishop et al. found a significant correlation between SLDJ height asymmetry and 505 performance (ρ = 0.65) (Bishop et al., 2022c), and Madruga-Parera et al. reported a significant association between SLLJ distance asymmetry and 180° CoD speed (r = 0.39) (Madruga-Parera et al., 2021). However, no studies have compared the relationship between single leg jump asymmetry and lateral CoD performance. This comparison would provide meaningful insights into the impact of inter-limb asymmetry on athletic performance.

The aims of this study were: 1) to examine whether basketball players exhibit significant inter-limb asymmetries in lateral CoD performance among basketball players using a specifically designed CoD in shuffle movement (CoDS) test; and 2) to assess the correlation and asymmetry direction agreement between CoDS, single leg jump and 505 test asymmetry score. The hypothesis of the present study was that basketball players will exhibit significant inter-limb asymmetries in CoD performance due to the nature of the movement. Additionally, it was hypothesized that the asymmetry observed in the CoDS test will significantly correlate with asymmetries in the single leg jump and 505 test scores, with consistent directional patterns across all tests.

## 2 Materials and methods

### 2.1 Study design

This descriptive study utilizes a correlational design to investigate: 1) differences and asymmetry in CoDS performance among basketball players, and 2) the relationship and directional agreement between asymmetry scores from CoDS, 505, and single leg jump tests. Data were collected from forty-two college basketball players, who completed three trials of the CoDS and 505 tests for each leg, with the fastest performance used for analysis. Additionally, participants performed single-leg countermovement jumps, lateral jumps, broad jumps, and drop jumps for both legs.

### 2.2 Participants

A total of 42 collegiate basketball players were recruited in this study, including 20 males and 22 females (Table 1). All athletes were from the university basketball team, participating in at least four regular basketball practices and two strength and conditioning sessions each week, with a minimum of 3 years of basketball training experience and prior participation in formal basketball competitions. Participants must have had no history of significant injuries in the 6 months prior to the study and should not suffer from chronic health conditions (e.g., cardiovascular diseases, diabetes) that could impair performance. The tests in this study were conducted prior to the start of the season and were approved by the local university ethics committee (Ethics approval number: ********).

**TABLE 1:** Participant characteristics (mean ± SD).

Variables	Male	Female	Total
Age (y)	20.35 ± 1.18	20.27 ± 1.24	20.30 ± 1.20
Height (cm)	184.50 ± 5.71	172.10 ± 6.02	178.30 ± 8.60
Body mass (kg)	83.23 ± 11.33	66.77 ± 9.77	75.30 ± 13.50
Training year (y)	6.40 ± 1.05	6.68 ± 1.43	6.55 ± 1.25
Guard	10	7	17
Forward	10	15	25

### 2.3 Procedures

Participants were tested on two different days, with a 48-h interval between each day. The first day included six trials of the 505 test and six trials of the CoDS test, while the second day involved four different single leg jump tests. Each participant underwent a specific warm-up routine (Turki et al., 2020), including 5 min of jogging and dynamic stretching for the lower body. After the warm-up, each test provided three practice trials.

For the analysis of jump and CoDS tests, we used My Jump two and CODTimer applications installed on an iPhone 13 Pro Max running iOS 18.0. These applications are designed to record video at 240 frames per second, with manual selection of the start and end of movements to calculate flight time (for jump tests) or sprint time (for CoD tests). A trained sports scientist with 2 years of experience using slow motion video applications recorded videos for each test using My Jump two and the CODTimer application for analysis.

### 2.4 Measurements

#### 2.4.1 Change of direction in shuffle test

**FIGURE 1:**
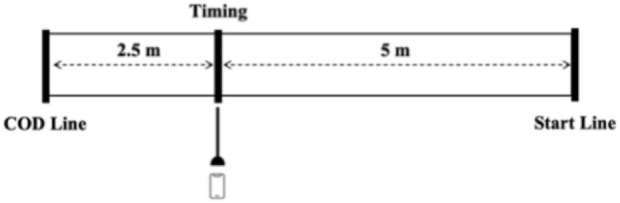
Structure and dimensions of the change of direction in shuffle test.

The CoDS test was designed based on the 505 test, and this test was performed on a basketball court. For convenience in setting up the testing area, and with prior studies indicating an average lateral movement distance of 2.28–4.24 m (Taylor et al., 2017), we set the acceleration shuffle distance to 5 m, with a timing point and a marker pole positioned at the 5 m mark to capture precise frames (Figure 1). The actual distance for CoD was 2.5 m. The reliability of using 2.5-m and 5-m distances in the shuffle test has been confirmed in a previous study (ICC = 0.90–0.96, CV = 1.5–2.4%) (Lyu et al., 2025a). Taking the left CoDS as an example. Participants started behind the starting line in a ready shuffle stance which asked as keep feet aligned with the shoulders, with knees flexed at approximately 60°, body slightly leaning forward, and center of gravity lowered (Lyu et al., 2024). Then shuffled to the left with maximum effort until their left foot crossed the CoD line. After completing the CoD, participants immediately shuffled to the right with maximum effort until they passed the timing point. Each participant completed three trials both on the left and right sides, with the trial order randomly assigned and a 3-min recovery interval provided between trials. The CODTimer, a validated tool for measuring CoD performance (Balsalobre-Fernández et al., 2019; Bishop et al., 2022b), was used for timing. The starting and ending frames were determined when the participant’s midline aligned with the marker pole at each endpoint.

#### 2.4.2 505 test

The 505 test was conducted following the procedures from previous studies (Bishop et al., 2022b). Consistent with the timing setup in the CoDS test, a timing point (Phone) and a marker pole were positioned at the 10 m mark to select the precise frames. This test was also performed on a basketball court. Participants started with their front foot positioned behind the starting line in a sprint stance. They sprinted through the timing point to reach the turning line, which was clearly marked on the floor. According to the trial requirements, participants placed either their left or right foot on or beyond the turning line before quickly pivoting and sprinting back through the timing point. Each participant completed three trials on both the left and right sides, with the trial order randomly assigned and a 3-min recovery interval provided between trials. The starting and ending frames were determined based on the alignment of the participant’s hip midpoint with the marker pole at each endpoint.

#### 2.4.3 Single leg countermovement jump test

Participants stood on one leg with their hands on their hips, preparing by performing a self-selected depth squat and then jumping as high as possible using the supporting leg. During the jump, the non-supporting leg remained slightly bent at the knee, with the foot positioned near the ankle of the supporting leg, no additional swinging of the non-supporting leg was allowed. The participants must maintain stable single-leg stance upon landing; any movement that violates the above requirements will result in the trial being deemed invalid. Each leg completed three SLCMJs, with a 60-s rest between trials. Any jump not meeting the movement criteria was marked invalid. The highest jump height from each leg was recorded for subsequent data analysis (Madruga-Parera et al., 2021). The SLCMJ was assessed using My Jump 2, a tool validated for accurately measuring jump height (Balsalobre-Fernández et al., 2015; Bishop et al., 2022b).

#### 2.4.4 Single leg lateral jump test

The SLLJ test measured lateral jump distance (in centimeters) using a standard measuring tape fixed to the ground. Participants began behind the 0 cm mark, lowered their bodies to a self-selected depth, and jumped as far laterally as possible, taking care not to land directly on the measuring tape. Hands remained on the hips throughout the jump. Given the increased difficulty associated with jumping in the frontal plane, participants were instructed to land with both feet simultaneously to ensure greater stability (Madruga-Parera et al., 2021). They were required to hold a stable landing position for 2 s. The distance was measured from the outer edge of the landing foot (the closest part to the 0 cm mark) to the take-off point. Each leg was tested three times with a 60-s rest between trials. The farthest jump distance for each leg was recorded for subsequent data analysis (Madruga-Parera et al., 2021).

#### 2.4.5 Single leg broad jump test

The SLBJ test measured horizontal jump distance (in centimeters) using a standard measuring tape fixed to the floor. Participants began behind the 0 cm starting line with their testing leg positioned for the jump. To initiate, they lowered their body to a self-selected depth, then jumped forward along the direction of the tape as far as possible, avoiding direct contact with the tape during landing. Hands remained on the hips throughout the jump. Participants were required to land stably on the same leg and hold this position for at least 2 s. The jump distance was measured from the heel of the landing foot. The non-jumping leg was kept slightly bent at the knee, with the foot positioned near the ankle of the jumping leg, and additional swinging of the non-jumping leg was not permitted. Any jump that did not meet these criteria was considered invalid and required retesting after a recovery period. Each leg was tested three times with a 60 s rest between trials, and the farthest jump distance for each leg was recorded for data analysis (Madruga-Parera et al., 2021).

#### 2.4.6 Single leg drop jump test

Each participant performed three SLDJ trials per leg, recorded in the same procedure as the SLCMJ trials. All jumps started from a height of 0.3 m. Participants stood on a box as instructed, with hands placed on the hips, landing on the same leg used for the takeoff, and then jumped as high as possible. The participants were asked to minimize ground contact time and maximize jump height. Each leg was tested three times with a 60 s rest between trials, and the highest jump height per leg was recorded for final analysis (Bishop et al., 2022b).

#### 2.4.7 Statistical analyses

Descriptive data are presented as means ± standard deviation (SD). The normality and homogeneity of variance of the data were assessed using the Shapiro-Wilk and Levene’s tests, respectively. Reliability among repetitions was evaluated using the intraclass correlation coefficient (ICC) for absolute agreement with a 95% confidence interval (95% CI), typical error of measurement (TEM), and coefficient of variation (CV) (Padulo et al., 2019). The magnitude of CV was classified as follows: poor (>10%), moderate (5%–10%), or good (<5%) (Banyard et al., 2017). The ICC was calculated using the ICC1,k model, which is appropriate for single raters with multiple measurements. The ICC was interpreted according to the standards of Koo and Li (Koo & Li, 2016), where values were defined as excellent (>0.9), good (0.75–0.9), moderate (0.5–0.75), and poor (<0.5). The dominant limb (D), which scored better, and the non-dominant limb (ND) were defined. The units of the CoDS and 505 test results are time, so the side with the shorter duration is considered the dominant side. Asymmetry for all tasks was calculated as follows: Asymmetry Score = (D–ND)/D * 100 (Madruga-Parera et al., 2021). Paired-sample t-tests were used to compare D and ND differences across all performances. Independent samples and the Mann–Whitney U test were used to assess the difference in the asymmetry scores the differences between male and female athletes. Effect size (ES) for paired comparisons was calculated as Cohen’s d and expressed with a 95% CI, defined as small (<0.2), moderate (0.2–0.5), and large (>0.8) mean differences (Cohen, 2013). Statistical significance was set at *p* < 0.05. The study (n = 42) was powered (with an alpha level of 0.05% and 80% power) to detect an effect size of approximately 0.5 (moderate effect). A minimum sample size of 34 participants was calculated, but 42 participants were ultimately included to enhance the robustness of the data (Faul et al., 2007). The Kappa coefficient was calculated to evaluate the level of consistency in asymmetry favoring the same side (asymmetry direction) (Bishop, 2021). Kappa values were interpreted as follows: ≤0 poor agreement, 0.01–0.20 slight agreement, 0.21–0.40 fair agreement, 0.41–0.60 moderate agreement, 0.61–0.80 substantial agreement, and 0.81–0.99 almost perfect agreement (Bishop et al., 2023). Spearman’s ρ correlations were used to examine relationships between performance and inter-limb asymmetry data. Correlation strength between test metrics was classified as trivial (≤0.1), small (0.1–0.3), moderate (0.3–0.5), large (0.5–0.7), very large (0.7–0.9), and nearly perfect (0.9–1.0) (Hopkins et al., 2009). All statistical analyses were conducted using IBM SPSS software version 26.0 (IBM, Armonk, NY, USA).

## 3 Results

Table 2 presents the data on change of direction, jump performance, asymmetry, and reliability. All tests exhibited a high level of reliability, with all ICC values exceeding 0.90 and CVs below 10%, indicating an acceptable level of measurement precision. The performance data for each test is normally distributed (all *p* < 0.05), whereas the asymmetry data does not follow a normal distribution. Asymmetry scores across all tests range from 2.54% to 10.0%. Significant differences are found between the dominant and non-dominant sides in all CoD and jump tests (all *p* < 0.01).

**TABLE 2:** Change of direction performance, asymmetry score, and test reliability data.

Test	Mean ± SD	ES (95% CI)	Asymmetry (%)	TEM (95% CI)	CV (%)	ICC (95% CI)
505-D (s)	2.59 ± 0.17*	−0.34 (−0.49, −0.18)	2.54 ± 2.28	0.05 (0.01, 0.16)	2.0 (0.1, 6.0)	0.94 (0.89, 0.96)
505-ND (s)	2.65 ± 0.18			0.05 (0.01, 0.14)	2.0 (0.1, 5.0)	0.95 (0.92, 0.97)
CoDS-D (s)	1.65 ± 0.13*	−0.49 (−0.66, −0.32)	3.66 ± 2.74	0.03 (0.01, 0.12)	3.0 (0.1, 7.0)	0.92 (0.86, 0.95)
CoDS-ND (s)	1.72 ± 0.15			0.05 (0.01, 0.17)	3.0 (0.1, 9.0)	0.93 (0.88, 0.96)
SLCMJ-D (cm)	18.43 ± 5.88*	0.33 (0.12, 0.54)	9.56 ± 7.90	0.96 (0.16, 2.96)	6.0 (1.0, 18.0)	0.98 (0.97, 0.99)
SLCMJ-ND (cm)	16.61 ± 5.22			1.05 (0.01, 2.72)	6.0 (0.1,13.0)	0.97 (0.94, 0.98)
SLLJ-D (cm)	149.45 ± 21.75*	0.25 (0.05, 0.45)	5.61 ± 3.42	5.38 (0.01, 12.25)	4.0 (0.1, 9.0)	0.91 (0.85, 0.98)
SLLJ-ND (cm)	140.90 ± 19.93			5.96 (1.25, 14.99)	4.0 (1.0, 12.0)	0.96 (0.93, 0.98)
SLBJ-D (cm)	170.69 ± 26.11*	0.30 (0.09, 0.51)	4.67 ± 4.14	5.53 (0.82, 15.63)	4.0 (0.1, 11.0)	0.98 (0.97, 0.99)
SLBJ-ND (cm)	162.83 ± 26.49			4.51 (0.01, 11.03)	3.0 (0.1, 7.0)	0.98 (0.97, 0.99)
SLDJ-D (cm)	18.73 ± 5.95*	0.31 (0.09, 0.52)	10.10 ± 6.88	1.09 (0.01, 3.14)	7.0 (0.1, 24.0)	0.97 (0.96, 0.98)
SLDJ-ND (cm)	16.91 ± 5.71			1.23 (0.01, 3.68)	8.0 (0.1, 34.0)	0.96 (0.93, 0.98)

Abbreviation: CoDS—change of direction in shuffle; SLCMJ—single leg countermovement jump; SLLJ—single leg lateral jump; SLBJ—single leg broad jump; SLDJ—single leg drop jump; D—dominant limb; ND—non-dominant limb; SD−standard deviation; TEM—typical error of measurement; CI—confidence intervals; CV—coefficient of variation; ICC—intraclass correlation coefficient; ES—effect size; cm—centimeters.

* = significantly different to the test on the non-dominant limb (p < 0.01).

**FIGURE 2:**
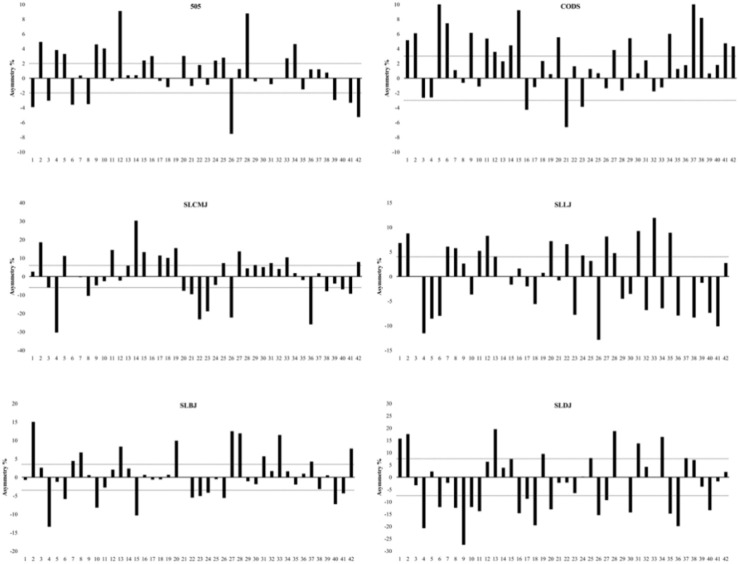
Individual inter-limb asymmetry data for change of direction and single leg jump performance. Note: The numbers on the horizontal axis represent information for each participant. Above 0 means the asymmetry favors the right limb, and below 0 means the asymmetry favors the left limb. The dotted lines represent the group threshold calculated from the pooled CV of the right and left limb or direction test scores (505 = 2.0%, CoDS = 3.0%, SLCMJ = 6.0%, SLLJ = 4.0%, SLBJ = 3.5%, SLDJ = 7.5%), which represents the average asymmetry score in the group. Abbreviations: CoDS—change of direction in shuffle; SLCMJ—single leg countermovement jump; SLLJ—single leg lateral jump; SLBJ—single leg broad jump; SLDJ—single leg drop jump.

The Kappa coefficients between CoDS asymmetry and single leg jump test asymmetry direction range from −0.07 to 0.22, indicating poor to slight agreement (Table 3). This suggests significant task-specific differences in dominant limb performance between CoD and single leg jump tasks (Figure 2). Table 4 shows the differences between female and male athletes in single leg jump and CoD performance, as well as the differences in asymmetry. Male athletes performed significantly better than female athletes in all performance measures (all *p* < 0.001), but there were no significant sex differences in asymmetry scores (all *p* > 0.05). The results of correlation analysis showed that neither 505 nor CoDS asymmetry is correlated with single leg jump asymmetry score (all *p* > 0.05). CoDS asymmetry score is significantly correlated with CoDS performance (ρ = 0.37, *p* < 0.05) but shows no significant correlation with 505 performance.

**TABLE 3:** Kappa coefficients and accompanying descriptors for levels of agreement describing how consistently asymmetry favored the same side in 505, CoDS and single leg jump test.

Test	505	CoDS	SLCMJ	SLLJ	SLBJ	SLDJ
505	1	0.15	0.07	0.17	0.23	0.30
CoDS		1	0.16	0.16	−0.07	0.22
SLCMJ			1	0.14	0.09	0.53
SLLJ				1	0.47	0.24
SLBJ					1	0.19
SLDJ						1

Abbreviation: CoDS—change of direction in shuffle; SLCMJ—single leg countermovement jump; SLLJ—single leg lateral jump; SLBJ—single leg broad jump; SLDJ—single leg drop jump.

**TABLE 4:** The differences in single leg jump and change of direction performance and asymmetry score between female and male athletes.

Test	Female	Male	*ES* (95% CI)	*p*
505-L (s)	2.71 ± 0.15*	2.51 ± 0.15	1.33 (0.73, 1.94)	<0.001
505-R (s)	2.74 ± 0.15*	2.51 ± 0.14	1.58 (0.97, 2.19)	<0.001
CoDS-L (s)	1.76 ± 0.12*	1.56 ± 0.09	1.87 (1.27, 2.50)	<0.001
CoDS -R (s)	1.80 ± 0.12*	1.60 ± 0.08	1.94 (1.34, 2.55)	<0.001
SLCMJ-L (cm)	13.56 ± 3.06*	21.88 ± 4.80	−2.09 (−3.97, −0.21)	<0.001
SLCMJ-R (cm)	14.01 ± 4.14*	21.39 ± 4.07	−1.80 (−3.49, −0.11)	<0.001
SLLJ-L (cm)	131.32 ± 14.82*	160.70 ± 17.80	−1.80 (−8.11, 4.51)	<0.001
SLLJ-R (cm)	132.59 ± 14.25*	158.75 ± 17.92	−1.63 (−7.25, 4.00)	<0.001
SLBJ-L (cm)	147.95 ± 16.83*	186.15 ± 20.22	−2.06 (−10.25, 6.13)	<0.001
SLBJ-R (cm)	148.45 ± 18.44*	188.20 ± 16.69	−2.25 (−10.78, 6.27)	<0.001
SLDJ-L (cm)	14.04 ± 3.39*	22.46 ± 5.12	−1.96 (−3.86, −0.06)	<0.001
SLDJ-R (cm)	13.50 ± 3.40*	22.09 ± 4.32	−2.22 (–4.16, −0.29)	<0.001
505 (%)	2.65 ± 2.14	2.42 ± 2.47	0.10 (−0.51, 0.71)	0.49
CoDS (%)	4.11 ± 2.81	3.15 ± 2.65	0.35 (−0.29, 0.99)	0.28
SLCMJ (%)	10.46 ± 8.89	8.57 ± 6.73	0.24 (−0.49, 0.97)	0.48
SLLJ (%)	4.80 ± 3.29	6.51 ± 3.42	−0.51 (−1.22, 0.20)	0.11
SLBJ (%)	4.66 ± 4.51	4.67 ± 3.81	−0.01 (−0.61, 0.60)	0.82
SLDJ (%)	11.22 ± 7.13	8.86 ± 6.55	0.34 (−0.44, 1.13)	0.31

Abbreviation: CoDS—change of direction in shuffle; SLCMJ—single leg countermovement jump; SLLJ—single leg lateral jump; SLBJ—single leg broad jump; SLDJ—single leg drop jump; L—left limb; R—right limb; ES—effect size; cm—centimeters. * = significantly different to the male group (p < 0.001).

## 4 Discussion

The purposes of this study were: 1) to examine whether there are differences and asymmetries in CoDS performance between limbs in basketball players, and 2) to assess the correlation and agreement of CoDS asymmetry with jump and 505 test asymmetry direction. Our results show that 1) basketball players exhibit differences in CoD performance between the limbs in both the forward-backward and lateral directions test; 2) no significant correlation and poor to slight agreement was found between asymmetry in CoDS, 505 and single leg jump performance. Based on this, the hypotheses of this study were accepted.

In Table 2 presents the reliability data for all tests, indicating that the CoDS is a reliable test. This reliability may stem from the fact that the test content is based on fundamental movements of basketball players, which could be equally applicable to athletes who frequently perform lateral movements. Previous studies have confirmed the reliability of using a mobile app to measure the 505 test (Bishop et al., 2022b), and our results further demonstrate that mobile apps are also reliable for assessing CoD performance during lateral movements. Furthermore, all the jump tests demonstrated high reliability (ICC ranged from 0.96 to 0.98, CV ranged from 2.0 to 8.0), which is consistent with the results of previous studies, highlighting the reliability of the single leg jump test and the reliability of using a phone app to measure jump height (Kons et al., 2021; Bishop et al., 2022b).

In the 505 test, there was a significant difference in performance between the dominant and non-dominant sides, with an asymmetry score of 2.54% ± 2.28% (ES = −0.34), consistent with previous research (Bishop et al., 2022b). Similarly, in the CoDS test, the performance difference between the dominant and non-dominant sides was significant, with an asymmetry score of 3.66% ± 2.74% (ES = −0.49), as expected. These results suggest that athletes are more proficient in performing rapid CoD on one side of their body, in both forward-backward and lateral direction movement. Although both tests showed asymmetry, the direction of asymmetry was entirely inconsistent, indicating that athletes rely on different movement patterns for two tests. The 505 test can be considered a forward-backward direction sprint-deceleration progression, athletes primarily rely on lower limb strength and coordination, closely linked to linear acceleration and deceleration abilities (Zhang et al., 2022). In contrast, lateral shuffle movement requires greater lateral mobility, with athletes depending on pelvis and core stability to achieve rapid side-to-side movements (Mohr and Federolf, 2022). From a biomechanical perspective, this asymmetry difference may stem from the fundamental distinctions in the kinematic and kinetic characteristics of the two tests. In the 505 test, athletes are required to accelerate forward and then quickly decelerate and change direction. This movement primarily involves eccentric and concentric contractions of the hip, knee, and ankle joints in the sagittal plane. The braking leg’s eccentric strength at the turning point plays a critical role in determining movement efficiency. In contrast, the CoDS shuffle test emphasizes lateral movement in the frontal plane, requiring greater control of ground reaction forces and horizontal pelvic stability. It particularly relies on the coordinated activation of the trunk and core muscles to maintain balance and fluidity during side-to-side motion. Furthermore, the two CoD tasks demand different neuromuscular control strategies. In the 505 test, the rapid recruitment of lower-limb muscles—especially in the dominant leg—directly impacts the speed of directional change. Meanwhile, the CoDS shuffle movement relies more on step control and coordination of trunk rotation. Due to these differences in movement mechanisms, the dominant side in each test may vary for the same athlete, resulting in inconsistent directions of asymmetry across tests. Directional asymmetries in movement may reflect imbalanced development of specific abilities during training (Dhahbi, 2025). In basketball, rapid lateral movement is crucial, especially in defense, where shuffle CoD ability is vital. To improve lateral movement and CoD abilities, particularly in response to the asymmetries observed in the CoDS test—which involves movement primarily in the frontal plane—training interventions should specifically target this movement direction. Incorporating frontal-plane plyometric exercises can help address these asymmetries and enhance lateral force production and neuromuscular control (McCormick et al., 2016). Additionally, specific lateral movement drills, such as lateral mini hurdle runs and lateral resistive runs, may directly improve the movement patterns and muscular coordination required for better performance in tasks like the CoDS (Kovacs, 2009). In addition, the asymmetry scores for both CoDS and the 505 test were no greater than 5%. Although previous studies have used thresholds of 10% or 15% as a reference for practitioners, it is necessary to establish different threshold standards for different tests (Pleša et al., 2024).

From a sex difference perspective, our results showed that male basketball players perform significantly better than female players in both CoD and single leg jump performance. However, all asymmetry score revealed no differences between male and female, which was consistent with previous studies and may suggest that asymmetry does not differ between sexes (Ding et al., 2024). The coexistence of performance disparities without corresponding differences in asymmetry may be attributable to the nature of asymmetry metrics, which quantify the relative performance balance between limbs rather than absolute ability. Although males generally exhibit greater muscular strength, power, and speed—leading to enhanced task performance—these advantages may be proportionally distributed across limbs, resulting in comparable asymmetry indices between sexes (Ding et al., 2024). In addition, the high intra-individual variability observed in asymmetry measures, characterized by large standard deviations relative to the mean, may obscure potential group-level differences. This could be due to the large intra-group variation in the relative percentage difference data, which has been explained in numerous empirical studies on the topic of asymmetry, this phenomenon has been well documented in the asymmetry literature and highlights the limitations of using group comparisons alone to draw inferences about asymmetry-related factors (Bishop et al., 2022d).

The 505 and CoDS asymmetry scores showed no significant correlation with single leg jump asymmetry scores, even though some single leg jump directions were similar to the movement directions in the CoD tests (e.g., SLBJ with 505, SLLJ with CoDS). This is consistent with previous research showing limited agreement in inter-limb asymmetry scores and direction between tasks and even different outcomes of the same task (Kozinc and Šarabon, 2020; Bishop et al., 2021; Pleša et al., 2024) This lack of association between asymmetry scores in CoD and jump test may be due to differences in movement patterns between CoD and jumps (Andreyo et al., 2024). Single leg jumps primarily rely on rapid lower limb power output, while the 505 and CoDS tests emphasize horizontal acceleration and deceleration demands and lateral movement demands. From the biomechanics perspective, these two tests involve significantly different movement patterns, especially lateral movements, which require greater stability from the pelvis, hips, and core to maintain balance and coordination. The characteristics of horizontal and lateral movements make them different from the vertical jump pattern, leading to a lack of significant correlation in asymmetry scores between the two types of tests. From a neuromuscular control mechanism perspective, different movement tasks rely on distinct neuromuscular control strategies. Single-leg jumps require athletes to quickly activate the explosive muscles in the lower limbs, demanding high neuromuscular coordination and rapid response capabilities. The neuromuscular control during the jump is typically focused on the fast activation of lower limb muscles and eccentric control to ensure stability and effectiveness of the action. Additionally, CoDS asymmetry score was significantly correlated with CoDS performance (r = 0.37, *p* < 0.05), though this correlation was primarily evident in the right limb CoDS performance. As shown in Figure 1, among the 42 athletes, 30 exhibited a right-sided bias in CoDS asymmetry. This suggests that coaches and practitioners should implement targeted assessment and training strategies to address asymmetry and promote balanced CoD abilities on both sides in basketball players, and specific asymmetry in athletic performance was also need to be measured in other sports (Kons et al., 2020; Madruga-Parera et al., 2021; Carvalho et al., 2024).

In this study, both CoD and jump tests were measured using a smart phone app. The smart phone is easy to operate and allows testing to be conducted anytime and anywhere, reducing reliance on expensive equipment and complex testing environments. This enables coaches to collect athlete data more efficiently. As a testing tool, the smart phone app greatly facilitates the monitoring of asymmetry in athletes during regular training (Balsalobre-Fernández et al., 2015; Romero-Franco et al., 2017; Bishop et al., 2022a; 2022b). For example, practitioners can regularly use mobile apps to test basketball players’ physical performance, such as jump, sprint, and agility tests. Practitioners can also conduct specific tests for athletes in different sports, such as the CoDS test we used for basketball players. The app can provide personalized comparisons and analyses of the athletes’ performance and this is meaningful for practitioners who seek convenience and low-cost solutions.

The limitations of this study are as follows. First, as this study used a smartphone application, the data only reflect outcome measurements and do not provide insights into task strategy. Second, since only within-session reliability of CoDS was assessed, conclusions about the test-retest reliability of CoDS are still uncertain. Future studies should examine the test-retest reliability of this test. Third, we only evaluated CoD ability in the left and right directions; however, basketball is a sport with multidirectional demands. Future research should explore CoD ability in additional directions to assess athletes’ shuffle and CoD abilities more comprehensively. In addition, the generalizability of the findings to athletes of different competitive levels and age groups is limited. Future studies should investigate athletes across a wider range of ages and competitive levels.

## 5 Conclusion

In conclusion, our study reveals significant asymmetries in CoD performance between the dominant and non-dominant limbs of basketball players, evident in both forward-backward and lateral movements. These findings highlight the need for targeted assessments aimed at improving lateral movement abilities. Given the convenience and practicality of mobile apps for testing, coaches and practitioners can leverage these tools to monitor and address asymmetries during training, ultimately enhancing athletes’ lateral movement and overall performance. Looking ahead, future research should examine the test-retest reliability of the CoDS and expand the evaluation to include multidirectional movements, providing a more comprehensive assessment of athletic capabilities.

## Data Availability

The original contributions presented in the study are included in the article/supplementary material, further inquiries can be directed to the corresponding author.
